# *trans*-Bis(quinoline-8-amine-κ^2^*N*,*N*′)bis­(1,1,3,3-tetra­cyano-2-meth­oxy­propenido-κ*N*)iron(II)

**DOI:** 10.1107/S2414314624012197

**Published:** 2024-12-20

**Authors:** Fatima Setifi, Zouaoui Setifi, Uwe Böhme, Mohammad Hadi Al-Douh, Achouak Satour

**Affiliations:** a Laboratoire de Chimie Ingénierie Moléculaire et Nanostructures (LCIMN) Université Ferhat Abbas Sétif 1 Sétif 19000 Algeria; b Départment de Technologie Faculté de Technologie Université 20 Août 1955-Skikda BP 26 Route d'El-Hadaiek Skikda 21000 Algeria; c Institut für Anorganische Chemie, Technische Universität Bergakademie Freiberg, Leipziger Str. 29, 09599 Freiberg, Germany; d Chemistry Department, Faculty of Science, Hadhramout University, Mukalla, Hadhramout, Yemen; Purdue University, USA

**Keywords:** crystal structure, iron(II) complex, quinoline-8-amine, 1,1,3,3-tetra­cyano-2-meth­oxy­propenide

## Abstract

The title compound, [Fe(C_8_H_3_N_4_O)_2_(C_9_H_8_N_2_)_2_], was synthesized solvothermally. The complex exhibits a distorted octa­hedral coordination geometry. The Fe^2+^ ion is located on an inversion centre. The octa­hedral FeN_6_ coordination sphere is composed of bidentate quinoline-8-amine in the equatorial sites while the axial sites are occupied by 1,1,3,3-tetra­cyano-2-meth­oxy­propenide anions. The crystal structure features hydrogen bonds parallel to the crystallographic *b* axis and parallel to (110).

## Abstract 2

[Fe(C_8_H_3_N_4_O)_2_(C_9_H_8_N_2_)_2_] exhibits a distorted octa­hedral coordination geometry. The coordination sphere of the iron(II) ion is composed of bidentate quinoline-8-amine in the equatorial sites while the axial sites are occupied by 1,1,3,3-tetra­cyano-2-meth­oxy­propenide anions.

## Graphical abstract

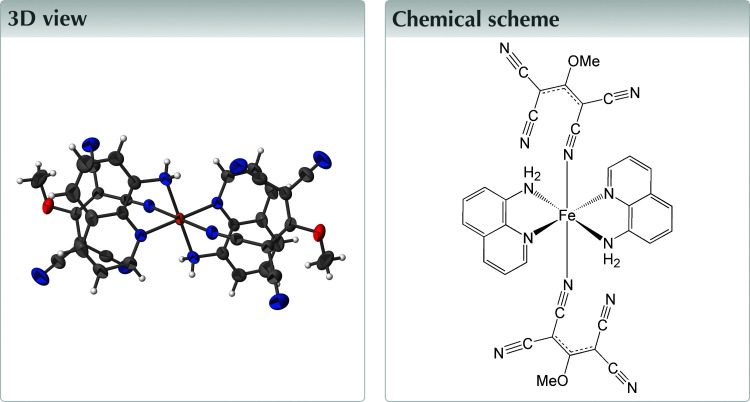

## Structure description

The well known spin-crossover (SCO) phenomenon can occur for some transition-metal complexes for which the metal ion is in a *d*^4^-, *d*^5^-, *d*^6^- or *d*^7^-configuration. The spin state can be switched between high-spin (HS) and low-spin (LS) states by an external perturbation such as temperature, pressure, magnetic field, or light irradiation (Benmansour *et al.*, 2010 ▸). In addition to the magnetic changes resulting from the spin state switching, this SCO behavior is accompanied by structural modifications and changes in the optical properties (color change), making the SCO system very promising for many potential applications such as the development of new generations of memory devices, sensors, displays, and organic light-emitting diodes (OLEDs) (Létard *et al.*, 2004 ▸; Halcrow, 2013 ▸).

Regarding the preparation of such SCO materials, our strategy is based on the use of cyano-carbanion ligands for designing these compounds. Taking into account their strong ability to adopt different bridging or non-bridging coordination modes (Addala *et al.*, 2015 ▸; Setifi *et al.*, 2013 ▸, 2014 ▸; Dmitrienko *et al.*, 2020 ▸), we have used them with other chelating ligands to explore their ability to generate a new series of Fe^II^–SCO complexes.

Continuing our study of spin-crossover 3*d*-metal complexes formed by polydentate and polynitrile units, we report here the synthesis and crystal structure of a new triclinic Fe^II^ complex, (I), which is the isostructural methoxy analogue of a previously described thio­methyl complex (Cuza *et al.*, 2021 ▸). This complex does not show any structural modifications at low temperature.

**Figure 1:**
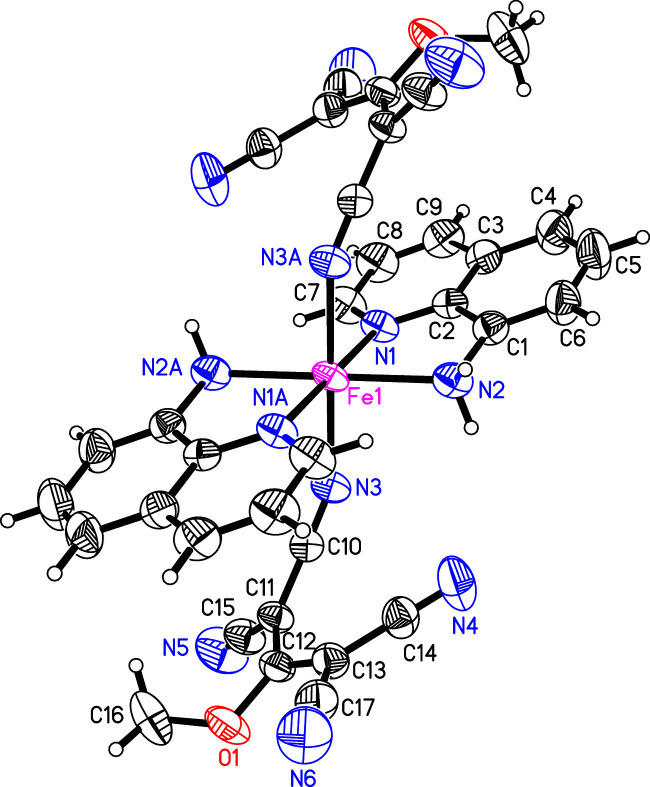
Mol­ecular structure of the title compound showing the atom-numbering scheme. Displacement ellipsoids are drawn with 50% probability level. Symmetry operator: (A) −*x* + 1, −*y* + 1, −*z* + 1.

The title compound shows an octa­hedral coordination around the Fe^2+^ ion. The positive charge of the central atom is compensated for by two 1,1,3,3-tetra­cyano-2-meth­oxy­propenido anions. Two mol­ecules of quinoline-8-amine are coordinated as well to the central atom. Thereby a neutral complex is generated, with the equal ligands in *trans*-position to each other. The iron atom is on a special position in the unit cell (*x* = ½, *y* = ½, *z* = ½) and the opposite ligands are generated by inversion around this position (Fig. 1 ▸). The *trans* angles in the complex are 180° due to the crystallographically imposed symmetry. However, there is substantial distortion from an ideal octa­hedron, as can be seen in the angle N3—Fe—N1 [94.72 (5)°] and the angle to the symmetry-equivalent nitro­gen atom N3^i^—Fe—N1 [symmetry code: (i) −*x* + 1, −*y* + 1, −*z* + 1] of 85.28 (5)°.

The iron atom is coordinated to six nitro­gen atoms with slightly different bond lengths (Table 1 ▸). The shortest bond is observed with N3 [2.152 (1) Å] from the anionic polynitrile ligand. The longest bond is to N2 [2.190 (2) Å], which is the NH_2_ group of the quinoline-8-amine. The bite angle of the quinoline-8-amine N1—Fe1—N2 is 77.62 (5)°, which is comparable to other complexes with this ligand (Setifi *et al.*, 2016 ▸; Cuza *et al.*, 2021 ▸).

The polynitrile anion 1,1,3,3-tetra­cyano-2-meth­oxy­propenide is distorted in itself. The plane of the atoms N3–C10–C11–C15–N5 forms a dihedral angle of 36.7 (1)° with the other di­cyano­methyl­ene group (N4–C14–C13–C17–N6). This is due to the coordination of the nitro­gen atom N3 to iron and the distribution of the negative charge in the anion. This type of distortion is often observed in polynitrile anions (Saadallah *et al.* 2022 ▸; Cuza *et al.*, 2021 ▸; Setifi, *et al.* 2017 ▸).

**Figure 2:**
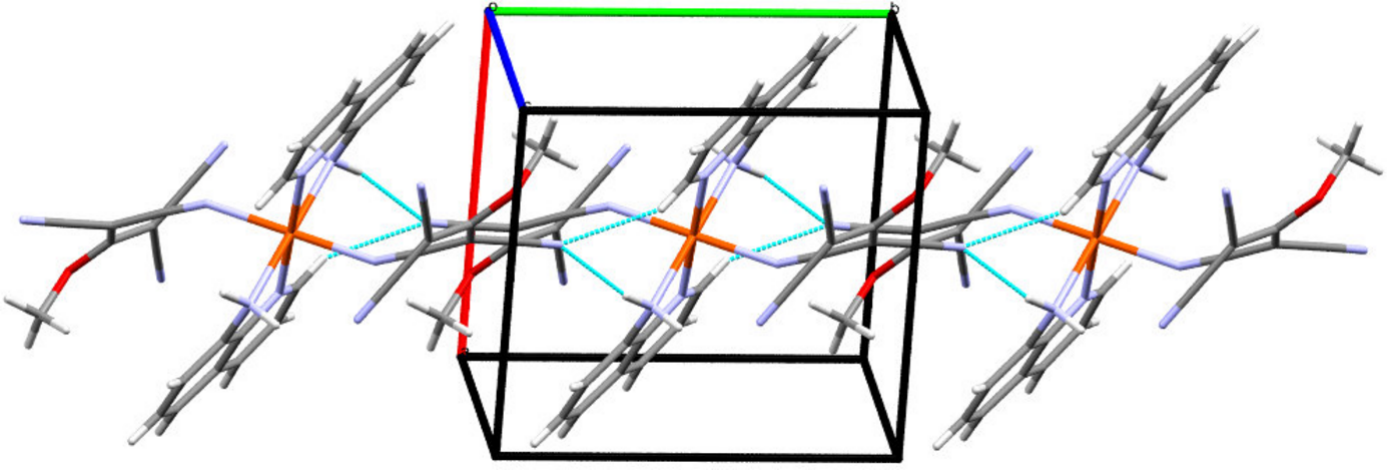
Partial packing diagram showing the N2—H1⋯N5 and C7—H7⋯N5 hydrogen-bonding inter­actions parallel to the crystallographic *b* axis.

**Figure 3:**
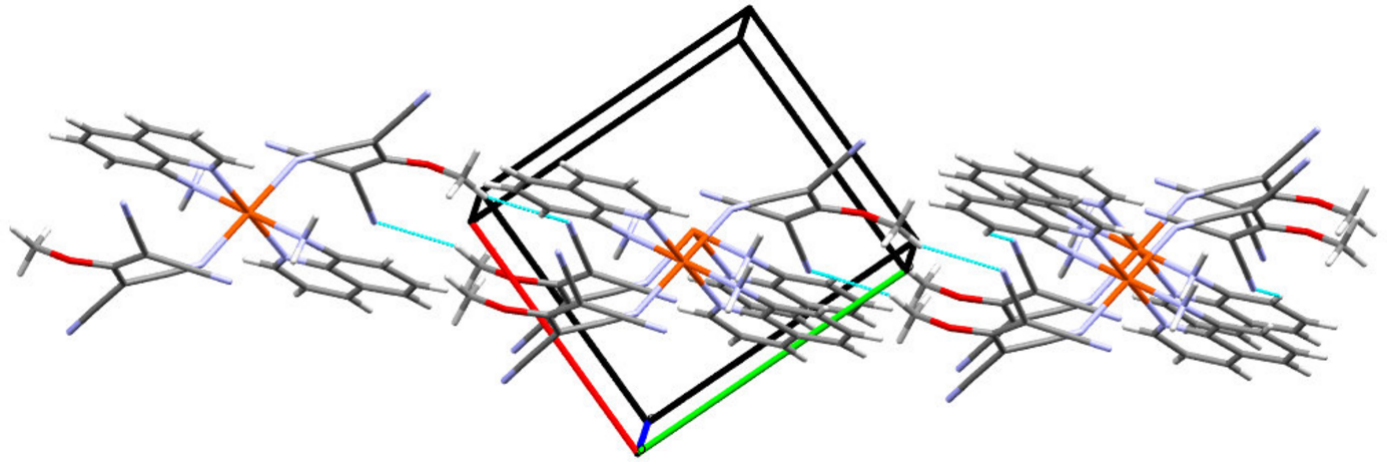
Partial packing diagram showing the C6—H6⋯N6 and C16—H16*A*⋯N6 hydrogen-bonding inter­actions parallel to (110).

The inter­molecular inter­actions are dominated by hydrogen bonds (Table 2 ▸). On the one hand there are bifurcated hydrogen bonds to N5 (N2—H1⋯N5^ii^ and C7—H7⋯N5^iii^), which form chains of mol­ecules parallel to the crystallographic *b* axis (Fig. 2 ▸). On the other hand N6 acts as a dual acceptor of hydrogen bonds (C6—H6⋯N6^iv^ and C16—H16⋯N6^v^), leading to the formation of layers parallel to (110) (Fig. 3 ▸; see Table 2 ▸ for numerical details). Both types of inter­actions combine to form a three-dimensional network of hydrogen bonds.

There is one isostructural iron complex in the literature (Cuza *et al.*, 2021 ▸). This complex has nearly the same composition as the title compound. It differs only in having a Me–S-group instead the Me–O-group in the title complex. The same publication features two more very similar complexes with a thio­ethyl- and a thio­propyl group in the polynitrile anions, respectively.

## Synthesis and crystallization

Compound (I) was prepared solvothermally from a mixture of iron(II) bis­(tetra­fluorido­borate) hexa­hydrate (34 mg, 0.1 mmol), 8-amino­quinoline (29 mg, 0.2 mmol) and potassium 1,1,3,3-tetra­cyano-2-meth­oxy­propenide (89 mg, 0.2 mmol) in a mixture of water/ethanol (4:1 *v*/*v*, 20 ml). This mixture was sealed in a Teflon-lined autoclave and held at 393 K for 2 d, and then cooled to ambient temperature at a rate of 10 K h^−1^ to give the product in form of yellow plates (yield 38%). Elemental analysis calculated for C_34_H_22_FeN_12_O_2_: C, 59.49; H, 3.23; N, 24.48%. Found: C, 60.73; H, 3.35; N, 24.17%. FT—IR (ATR, cm^−1^): 2187 (*vs*, tcnoMe).

## Refinement

Crystal data, data collection and structure refinement details are summarized in Table 3 ▸.

## Supplementary Material

Crystal structure: contains datablock(s) I. DOI: 10.1107/S2414314624012197/zl4078sup1.cif

Structure factors: contains datablock(s) I. DOI: 10.1107/S2414314624012197/zl4078Isup2.hkl

CCDC reference: 2411351

Additional supporting information:  crystallographic information; 3D view; checkCIF report

## Figures and Tables

**Table 1:** Selected geometric parameters (Å, °)

Fe1—N1	2.167 (1)	Fe1—N3	2.152 (1)
Fe1—N2	2.190 (2)		
			
N1^i^—Fe1—N1	180.0	N3—Fe1—N2	93.15 (6)
N2^i^—Fe1—N2	180.0	N3—Fe1—N2^i^	86.85 (6)
N3^i^—Fe1—N3	180.0	N1—Fe1—N2	77.62 (5)
N3—Fe1—N1	94.72 (5)	N1^i^—Fe1—N2	102.38 (5)
N3^i^—Fe1—N1	85.28 (5)		

Symmetry code: (i) 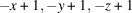.

**Table 2:** Hydrogen-bond geometry (Å, °)

*D*—H⋯*A*	*D*—H	H⋯*A*	*D*⋯*A*	*D*—H⋯*A*
N2—H1⋯N5^ii^	0.91 (3)	2.26 (3)	3.138 (3)	162 (2)
C7—H7⋯N5^iii^	0.93	2.58	3.392 (3)	146
C6—H6⋯N6^iv^	0.93	2.58	3.401 (3)	148
C16—H16*A*⋯N6^v^	0.96	2.64	3.355 (3)	132

Symmetry codes: (ii) 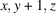; (iii) 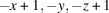; (iv) 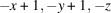; (v) 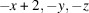.

**Table 3:** Experimental details

Crystal data	
Chemical formula	[Fe(C_8_H_3_N_4_O)_2_(C_9_H_8_N_2_)_2_]
*M*_r_	686.48
Crystal system, space group	Triclinic, *P* 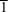
Temperature (K)	299
*a*, *b*, *c* (Å)	8.3617 (5), 9.8067 (6), 10.1251 (5)
α, β, γ (°)	100.206 (3), 90.276 (3), 90.500 (3)
*V* (Å^3^)	817.08 (8)
*Z*	1
Radiation type	Mo *K*α
μ (mm^−1^)	0.51
Crystal size (mm)	0.21 × 0.18 × 0.05

Data collection	
Diffractometer	Bruker D8 VENTURE Duo
Absorption correction	Multi-scan (*SADABS*; Krause *et al.*, 2015 ▸)
*T*_min_, *T*_max_	0.675, 0.745
No. of measured, independent and observed [*I* > 2σ(*I*)] reflections	41760, 4640, 3909
*R*_int_	0.048
(sin θ/λ)_max_ (Å^−1^)	0.697

Refinement	
*R*[*F*^2^ > 2σ(*F*^2^)], *wR*(*F*^2^), *S*	0.039, 0.104, 1.06
No. of reflections	4640
No. of parameters	232
H-atom treatment	H atoms treated by a mixture of independent and constrained refinement
Δρ_max_, Δρ_min_ (e Å^−3^)	0.33, −0.20

Computer programs: *APEX4* and *SAINT* (Bruker, 2021 ▸), *SHELXT* (Sheldrick, 2015*a* ▸), *SHELXL2017/1* (Sheldrick, 2015*b* ▸), *ORTEP-3 for Windows* (Farrugia, 2012 ▸), *Mercury* (Macrae *et al.*, 2020 ▸) and *publCIF* (Westrip, 2010 ▸).

## full crystallographic data

### trans-Bis(quinoline-8-amine-κ2N,N')bis(1,1,3,3-tetracyano-2-methoxypropenido-κN)iron(II) Crystal data

**Table:**

[Fe(C_8_H_3_N_4_O)_2_(C_9_H_8_N_2_)_2_]	*Z* = 1
*M**_r_* = 686.48	*F*(000) = 352
Triclinic, *P*1	*D*_x_ = 1.395 Mg m^−^^3^
*a* = 8.3617 (5) Å	Mo *K*α radiation, λ = 0.71073 Å
*b* = 9.8067 (6) Å	Cell parameters from 3704 reflections
*c* = 10.1251 (5) Å	θ = 2.9–28.5°
α = 100.206 (3)°	µ = 0.51 mm^−^^1^
β = 90.276 (3)°	*T* = 299 K
γ = 90.500 (3)°	Plate, yellow
*V* = 817.08 (8) Å^3^	0.21 × 0.18 × 0.05 mm

### trans-Bis(quinoline-8-amine-κ2N,N')bis(1,1,3,3-tetracyano-2-methoxypropenido-κN)iron(II) Data collection

**Table:**

Bruker D8 VENTURE Duo diffractometer	4640 independent reflections
Radiation source: sealed tube	3909 reflections with *I* > 2σ(*I*)
TRIUMPH graphite monochromator	*R*_int_ = 0.048
ω and φ scans	θ_max_ = 29.7°, θ_min_ = 2.4°
Absorption correction: multi-scan (SADABS; Krause *et al.*, 2015)	*h* = −11→11
*T*_min_ = 0.675, *T*_max_ = 0.745	*k* = −13→13
41760 measured reflections	*l* = −14→14

### trans-Bis(quinoline-8-amine-κ2N,N')bis(1,1,3,3-tetracyano-2-methoxypropenido-κN)iron(II) Refinement

**Table:**

Refinement on *F*^2^	Primary atom site location: structure-invariant direct methods
Least-squares matrix: full	Secondary atom site location: difference Fourier map
*R*[*F*^2^ > 2σ(*F*^2^)] = 0.039	Hydrogen site location: mixed
*wR*(*F*^2^) = 0.104	H atoms treated by a mixture of independent and constrained refinement
*S* = 1.06	*w* = 1/[σ^2^(*F*_o_^2^) + (0.0412*P*)^2^ + 0.3359*P*] where *P* = (*F*_o_^2^ + 2*F*_c_^2^)/3
4640 reflections	(Δ/σ)_max_ < 0.001
232 parameters	Δρ_max_ = 0.33 e Å^−^^3^
0 restraints	Δρ_min_ = −0.20 e Å^−^^3^

### trans-Bis(quinoline-8-amine-κ2N,N')bis(1,1,3,3-tetracyano-2-methoxypropenido-κN)iron(II) Special details

**Table:**

Geometry. All esds (except the esd in the dihedral angle between two l.s. planes) are estimated using the full covariance matrix. The cell esds are taken into account individually in the estimation of esds in distances, angles and torsion angles; correlations between esds in cell parameters are only used when they are defined by crystal symmetry. An approximate (isotropic) treatment of cell esds is used for estimating esds involving l.s. planes.
Refinement. All non-hydrogen atoms were refined anisotropically. The hydrogen atoms at N2 have been localized from residual electron density peaks and were freely refined. All other hydrogen atoms were placed in idealized positions and refined with a riding model.

### trans-Bis(quinoline-8-amine-κ2N,N')bis(1,1,3,3-tetracyano-2-methoxypropenido-κN)iron(II) Fractional atomic coordinates and isotropic or equivalent isotropic displacement parameters (Å^2^)

**Table:**

	*x*	*y*	*z*	*U*_iso_*/*U*_eq_	
Fe1	0.500000	0.500000	0.500000	0.03405 (10)	
O1	0.74346 (19)	0.00197 (15)	0.05960 (15)	0.0609 (4)	
N1	0.29013 (17)	0.50285 (13)	0.62516 (13)	0.0378 (3)	
N2	0.32247 (19)	0.59938 (16)	0.38964 (14)	0.0420 (3)	
H1	0.364 (3)	0.671 (3)	0.354 (2)	0.067 (7)*	
H2	0.283 (3)	0.538 (3)	0.320 (3)	0.073 (7)*	
N3	0.45064 (17)	0.29722 (14)	0.38569 (14)	0.0406 (3)	
N4	0.3677 (2)	0.3604 (2)	0.0862 (2)	0.0707 (5)	
N5	0.5201 (3)	−0.14868 (19)	0.3218 (2)	0.0780 (6)	
N6	0.8157 (3)	0.2527 (3)	−0.1261 (2)	0.0810 (6)	
C1	0.1911 (2)	0.64840 (17)	0.47750 (16)	0.0391 (3)	
C2	0.17703 (18)	0.59330 (15)	0.59786 (15)	0.0341 (3)	
C3	0.04890 (19)	0.6362 (2)	0.68605 (17)	0.0432 (4)	
C4	−0.0580 (2)	0.7348 (3)	0.6558 (2)	0.0623 (6)	
H4	−0.140959	0.765119	0.714225	0.075*	
C5	−0.0405 (3)	0.7861 (3)	0.5409 (2)	0.0705 (6)	
H5	−0.113006	0.851238	0.521376	0.085*	
C6	0.0837 (2)	0.7439 (2)	0.4505 (2)	0.0545 (5)	
H6	0.092832	0.780996	0.372447	0.065*	
C7	0.2749 (2)	0.45015 (19)	0.73573 (18)	0.0482 (4)	
H7	0.351765	0.388237	0.754634	0.058*	
C8	0.1491 (2)	0.4828 (2)	0.82583 (19)	0.0536 (5)	
H8	0.142130	0.441280	0.901400	0.064*	
C9	0.0377 (2)	0.5748 (2)	0.80278 (18)	0.0524 (5)	
H9	−0.045781	0.597725	0.862782	0.063*	
C10	0.49612 (18)	0.19840 (15)	0.32012 (15)	0.0337 (3)	
C11	0.5592 (2)	0.08061 (15)	0.23830 (16)	0.0380 (3)	
C12	0.6374 (2)	0.09521 (16)	0.11809 (16)	0.0387 (3)	
C13	0.6126 (2)	0.20359 (18)	0.04875 (16)	0.0410 (3)	
C14	0.4770 (2)	0.2904 (2)	0.06994 (18)	0.0466 (4)	
C15	0.5392 (3)	−0.04715 (18)	0.28338 (19)	0.0499 (4)	
C16	0.8475 (3)	−0.0676 (3)	0.1383 (3)	0.0799 (8)	
H16A	0.947226	−0.086625	0.092450	0.120*	
H16B	0.798397	−0.153116	0.151162	0.120*	
H16C	0.866451	−0.009761	0.223891	0.120*	
C17	0.7249 (2)	0.2298 (2)	−0.04895 (19)	0.0537 (5)	

### trans-Bis(quinoline-8-amine-κ2N,N')bis(1,1,3,3-tetracyano-2-methoxypropenido-κN)iron(II) Atomic displacement parameters (Å^2^)

**Table:**

	*U*^11^	*U*^22^	*U*^33^	*U*^12^	*U*^13^	*U*^23^
Fe1	0.03897 (18)	0.02907 (15)	0.03390 (16)	0.00370 (11)	0.01334 (12)	0.00447 (11)
O1	0.0683 (9)	0.0512 (8)	0.0610 (8)	0.0250 (7)	0.0186 (7)	0.0023 (6)
N1	0.0448 (7)	0.0317 (6)	0.0375 (6)	0.0003 (5)	0.0127 (5)	0.0076 (5)
N2	0.0508 (8)	0.0426 (7)	0.0339 (7)	0.0019 (6)	0.0116 (6)	0.0096 (6)
N3	0.0466 (8)	0.0347 (7)	0.0396 (7)	−0.0003 (6)	0.0106 (6)	0.0039 (5)
N4	0.0607 (11)	0.0799 (13)	0.0795 (13)	0.0280 (10)	0.0094 (10)	0.0342 (11)
N5	0.1082 (17)	0.0418 (9)	0.0890 (14)	0.0018 (10)	0.0172 (12)	0.0246 (9)
N6	0.0728 (13)	0.1186 (19)	0.0583 (11)	0.0074 (12)	0.0229 (10)	0.0329 (12)
C1	0.0381 (8)	0.0429 (8)	0.0358 (7)	−0.0013 (6)	0.0036 (6)	0.0058 (6)
C2	0.0333 (7)	0.0349 (7)	0.0328 (7)	−0.0054 (6)	0.0060 (5)	0.0024 (5)
C3	0.0295 (7)	0.0561 (10)	0.0414 (8)	−0.0036 (7)	0.0061 (6)	0.0010 (7)
C4	0.0373 (9)	0.0915 (16)	0.0569 (11)	0.0170 (10)	0.0080 (8)	0.0089 (11)
C5	0.0534 (12)	0.0933 (17)	0.0687 (14)	0.0300 (12)	0.0032 (10)	0.0233 (12)
C6	0.0516 (11)	0.0684 (12)	0.0472 (10)	0.0136 (9)	0.0014 (8)	0.0197 (9)
C7	0.0590 (11)	0.0426 (9)	0.0466 (9)	0.0034 (8)	0.0165 (8)	0.0171 (7)
C8	0.0596 (11)	0.0628 (12)	0.0421 (9)	−0.0025 (9)	0.0184 (8)	0.0188 (8)
C9	0.0417 (9)	0.0722 (13)	0.0425 (9)	−0.0065 (9)	0.0155 (7)	0.0076 (8)
C10	0.0354 (7)	0.0315 (7)	0.0348 (7)	−0.0034 (5)	0.0028 (6)	0.0079 (5)
C11	0.0464 (9)	0.0274 (7)	0.0400 (8)	0.0023 (6)	0.0037 (6)	0.0049 (6)
C12	0.0414 (8)	0.0334 (7)	0.0393 (8)	0.0051 (6)	0.0038 (6)	0.0002 (6)
C13	0.0417 (8)	0.0460 (9)	0.0358 (7)	0.0048 (7)	0.0056 (6)	0.0083 (6)
C14	0.0478 (10)	0.0520 (10)	0.0439 (9)	0.0062 (8)	0.0030 (7)	0.0184 (7)
C15	0.0641 (12)	0.0330 (8)	0.0531 (10)	0.0024 (8)	0.0063 (9)	0.0086 (7)
C16	0.0629 (14)	0.0688 (15)	0.110 (2)	0.0318 (12)	0.0115 (14)	0.0208 (14)
C17	0.0528 (11)	0.0691 (13)	0.0415 (9)	0.0055 (9)	0.0068 (8)	0.0154 (8)

### trans-Bis(quinoline-8-amine-κ2N,N')bis(1,1,3,3-tetracyano-2-methoxypropenido-κN)iron(II) Geometric parameters (Å, º)

**Table:**

Fe1—N1	2.167 (1)	C3—C9	1.422 (3)
Fe1—N2	2.190 (2)	C4—C5	1.355 (3)
Fe1—N3	2.152 (1)	C4—H4	0.9300
Fe1—N1^i^	2.167 (1)	C5—C6	1.404 (3)
Fe1—N2^i^	2.190 (2)	C5—H5	0.9300
Fe1—N3^i^	2.152 (1)	C6—H6	0.9300
O1—C12	1.3421 (19)	C7—C8	1.398 (2)
O1—C16	1.433 (3)	C7—H7	0.9300
N1—C7	1.320 (2)	C8—C9	1.350 (3)
N1—C2	1.363 (2)	C8—H8	0.9300
N2—C1	1.448 (2)	C9—H9	0.9300
N2—H1	0.91 (3)	C10—C11	1.405 (2)
N2—H2	0.90 (3)	C11—C12	1.413 (2)
N3—C10	1.143 (2)	C11—C15	1.416 (2)
N4—C14	1.143 (2)	C12—C13	1.390 (2)
N5—C15	1.142 (2)	C13—C14	1.418 (2)
N6—C17	1.141 (3)	C13—C17	1.422 (2)
C1—C6	1.364 (3)	C16—H16A	0.9600
C1—C2	1.423 (2)	C16—H16B	0.9600
C2—C3	1.416 (2)	C16—H16C	0.9600
C3—C4	1.395 (3)		
			
N1^i^—Fe1—N1	180.0	C5—C4—H4	120.1
N2^i^—Fe1—N2	180.0	C3—C4—H4	120.1
N3^i^—Fe1—N3	180.0	C4—C5—C6	122.0 (2)
N3—Fe1—N1	94.72 (5)	C4—C5—H5	119.0
N3^i^—Fe1—N1	85.28 (5)	C6—C5—H5	119.0
N3—Fe1—N2	93.15 (6)	C1—C6—C5	119.83 (18)
N3—Fe1—N2^i^	86.85 (6)	C1—C6—H6	120.1
N1—Fe1—N2	77.62 (5)	C5—C6—H6	120.1
N1^i^—Fe1—N2	102.38 (5)	N1—C7—C8	123.26 (18)
N3^i^—Fe1—N1^i^	94.72 (5)	N1—C7—H7	118.4
N3—Fe1—N1^i^	85.28 (5)	C8—C7—H7	118.4
N3^i^—Fe1—N2^i^	93.15 (6)	C9—C8—C7	119.78 (17)
N1^i^—Fe1—N2^i^	77.62 (5)	C9—C8—H8	120.1
N1—Fe1—N2^i^	102.38 (5)	C7—C8—H8	120.1
N3^i^—Fe1—N2	86.85 (6)	C8—C9—C3	119.47 (16)
C12—O1—C16	121.08 (17)	C8—C9—H9	120.3
C7—N1—C2	118.11 (14)	C3—C9—H9	120.3
C7—N1—Fe1	127.48 (12)	N3—C10—C11	177.07 (17)
C2—N1—Fe1	113.00 (9)	C10—C11—C12	119.28 (14)
C1—N2—Fe1	109.58 (10)	C10—C11—C15	116.66 (15)
C1—N2—H1	109.7 (15)	C12—C11—C15	124.06 (15)
Fe1—N2—H1	113.0 (15)	O1—C12—C13	113.69 (15)
C1—N2—H2	108.1 (17)	O1—C12—C11	121.78 (15)
Fe1—N2—H2	110.5 (16)	C13—C12—C11	124.53 (14)
H1—N2—H2	106 (2)	C12—C13—C14	122.64 (15)
C10—N3—Fe1	149.11 (13)	C12—C13—C17	119.91 (16)
C6—C1—C2	119.65 (16)	C14—C13—C17	117.45 (16)
C6—C1—N2	123.29 (15)	N4—C14—C13	179.6 (2)
C2—C1—N2	117.06 (14)	N5—C15—C11	178.3 (2)
N1—C2—C3	122.40 (14)	O1—C16—H16A	109.5
N1—C2—C1	118.37 (13)	O1—C16—H16B	109.5
C3—C2—C1	119.22 (15)	H16A—C16—H16B	109.5
C4—C3—C2	119.59 (17)	O1—C16—H16C	109.5
C4—C3—C9	123.51 (17)	H16A—C16—H16C	109.5
C2—C3—C9	116.91 (17)	H16B—C16—H16C	109.5
C5—C4—C3	119.73 (19)	N6—C17—C13	178.9 (3)
			
Fe1—N2—C1—C6	162.71 (16)	N2—C1—C6—C5	180.0 (2)
Fe1—N2—C1—C2	−16.71 (18)	C4—C5—C6—C1	0.0 (4)
C7—N1—C2—C3	2.6 (2)	C2—N1—C7—C8	−0.1 (3)
Fe1—N1—C2—C3	−164.88 (12)	Fe1—N1—C7—C8	165.26 (15)
C7—N1—C2—C1	−178.81 (15)	N1—C7—C8—C9	−1.5 (3)
Fe1—N1—C2—C1	13.75 (17)	C7—C8—C9—C3	0.7 (3)
C6—C1—C2—N1	−177.05 (16)	C4—C3—C9—C8	−178.2 (2)
N2—C1—C2—N1	2.4 (2)	C2—C3—C9—C8	1.5 (3)
C6—C1—C2—C3	1.6 (2)	C16—O1—C12—C13	145.8 (2)
N2—C1—C2—C3	−178.93 (15)	C16—O1—C12—C11	−34.8 (3)
N1—C2—C3—C4	176.54 (17)	C10—C11—C12—O1	157.48 (16)
C1—C2—C3—C4	−2.1 (2)	C15—C11—C12—O1	−22.5 (3)
N1—C2—C3—C9	−3.3 (2)	C10—C11—C12—C13	−23.2 (3)
C1—C2—C3—C9	178.12 (15)	C15—C11—C12—C13	156.82 (19)
C2—C3—C4—C5	1.5 (3)	O1—C12—C13—C14	161.86 (17)
C9—C3—C4—C5	−178.7 (2)	C11—C12—C13—C14	−17.5 (3)
C3—C4—C5—C6	−0.5 (4)	O1—C12—C13—C17	−17.4 (2)
C2—C1—C6—C5	−0.6 (3)	C11—C12—C13—C17	163.18 (17)

Symmetry code: (i) −*x*+1, −*y*+1, −*z*+1.

### trans-Bis(quinoline-8-amine-κ2N,N')bis(1,1,3,3-tetracyano-2-methoxypropenido-κN)iron(II) Hydrogen-bond geometry (Å, º)

**Table:**

*D*—H···*A*	*D*—H	H···*A*	*D*···*A*	*D*—H···*A*
N2—H1···N5^ii^	0.91 (3)	2.26 (3)	3.138 (3)	162 (2)
C7—H7···N5^iii^	0.93	2.58	3.392 (3)	146
C6—H6···N6^iv^	0.93	2.58	3.401 (3)	148
C16—H16*A*···N6^v^	0.96	2.64	3.355 (3)	132

Symmetry codes: (ii) *x*, *y*+1, *z*; (iii) −*x*+1, −*y*, −*z*+1; (iv) −*x*+1, −*y*+1, −*z*; (v) −*x*+2, −*y*, −*z*.
